# DNA methylation epigenotypes in breast cancer molecular subtypes

**DOI:** 10.1186/bcr2721

**Published:** 2010-09-29

**Authors:** Naiara G Bediaga, Amelia Acha-Sagredo, Isabel Guerra, Amparo Viguri, Carmen Albaina, Irune Ruiz Diaz, Ricardo Rezola, María Jesus Alberdi, Joaquín Dopazo, David Montaner, Mertxe de Renobales, Agustín F Fernández, John K Field, Mario F Fraga, Triantafillos Liloglou, Marian M de Pancorbo

**Affiliations:** 1 BIOMICs Research Group, Centro de Investigacion y Estudios Avanzados 'Lucio Lascaray', University of the Basque Country UPV/EHU, Miguel de Unamuno 3,1006, Vitoria-Gazteiz, Spain; 2 Oral Medicine and Pathology Unit, Faculty of Medicine and Dentistry, University of the Basque Country UPV/EH, Barrio Sarriena s/n. E-48940 Leioa, Vizcaya, Spain; 3 Service of Anatomic Pathology, Hospital Txagorritxu, C/Jose Achotegui s/n, E-01009 Vitoria-Gasteiz, Alava, Spain; 4 Service of Anatomic Pathology, Hospital Donostia, Paseo Beguiristain 107-115, E-20014 San Sebastian, Guipuzcoa, Spain; 5 Service of Anatomic Pathology, Instituto Oncologico, C/Aldakoenea 44, E-20012 San Sebastian, Spain; 6 Department of Bioinformatics and Genomics, Centro de Investigación Principe Felipe, Avda. Autopista del Saler, 164, E-6012 Valencia, Spain; 7 Molecular Biology and Biochemistry, Faculty of Pharmacy, University of the Basque Country UPV/EHU, Paseo de la Universidad 7, E-01006 Vitoria-Gasteiz, Spain; 8 Cancer Epigenetics Laboratory, Instituto Universitario de Oncologia del Principado de Asturias (IUOPA), HUCA, Universidad de Oviedo, E- 33006 Oviedo, Spain; 9 Liverpool CR-UK Cancer Research Centre, University of Liverpool Cancer, School of Cancer Studies, 200 London Road, Liverpool L3 9TA, UK; 10 Department of Immunology and Oncology, National Center for Biotechnology, CNB-CSIC, Cantoblanco, E-28049 Madrid, Spain

## Abstract

**Introduction:**

Identification of gene expression-based breast cancer subtypes is considered a critical means of prognostication. Genetic mutations along with epigenetic alterations contribute to gene-expression changes occurring in breast cancer. So far, these epigenetic contributions to sporadic breast cancer subtypes have not been well characterized, and only a limited understanding exists of the epigenetic mechanisms affected in those particular breast cancer subtypes. The present study was undertaken to dissect the breast cancer methylome and to deliver specific epigenotypes associated with particular breast cancer subtypes.

**Methods:**

By using a microarray approach, we analyzed DNA methylation in regulatory regions of 806 cancer-related genes in 28 breast cancer paired samples. We subsequently performed substantial technical and biologic validation by pyrosequencing, investigating the top qualifying 19 CpG regions in independent cohorts encompassing 47 basal-like, 44 ERBB2+ overexpressing, 48 luminal A, and 48 luminal B paired breast cancer/adjacent tissues. With the all-subset selection method, we identified the most subtype-predictive methylation profiles in multivariable logistic regression analysis.

**Results:**

The approach efficiently recognized 15 individual CpG loci differentially methylated in breast cancer tumor subtypes. We further identified novel subtype-specific epigenotypes that clearly demonstrate the differences in the methylation profiles of basal-like and human epidermal growth factor 2 (HER2)-overexpressing tumors.

**Conclusions:**

Our results provide evidence that well-defined DNA methylation profiles enable breast cancer subtype prediction and support the utilization of this biomarker for prognostication and therapeutic stratification of patients with breast cancer.

## Introduction

Breast cancer is a heterogeneous disease, both biologically and clinically. The molecular background behind breast cancer progression is not well understood, but is associated with the accumulation of genetic aberrations, leading to widespread gene-expression changes in breast tumor cells. Consistent with this is the presence of at least four major breast cancer subtypes with distinct expression patterns and clinical outcomes. These subtypes are termed basal-like, ERBB2+, luminal B, and luminal A [1,2]. Basal-like and ERBB2+ subtypes are hormone-receptor negative and have poor prognoses. In contrast, luminal breast cancers are characterized by the expression of ER-associated genes, with luminal B tumors having poorer outcomes than luminal A tumors. Although this gene expression-based approach has proven to add significant prognostic and predictive value to pathologic staging, histologic grade, and standard clinical molecular markers [3], the high cost of expression profiling and the molecular instability of the mRNA transcripts has limited its incorporation into clinical settings, and therefore, expression-based breast cancer classification has not become a standardized method in the general practice [4]. Thus, although breast cancer stratification by gene expression is still considered the gold standard, an urgent need exists for well-defined biomarker panels allowing breast cancer subtyping in clinical diagnostics.

Epigenetic alterations such as aberrations in DNA methylation, microRNA patterns, and post-translational modifications of histones are common molecular abnormalities in cancer [5]. Furthermore, many studies suggest that epigenetic changes are involved in the earliest phases of tumorigenesis, and that they may predispose stem/progenitor cells to subsequent genetic and epigenetic changes involved in tumor promotion [6]. Cancer-related disruption of the DNA methylome involves global genomic hypomethylation and regional hypermethylation of cytosine-phosphate-guanine (CpG) islands. The first can lead to chromosomal instability [7], whereas the second is frequently associated with promoters of tumor-suppressor genes, resulting in their transcriptional silencing. Both such alterations have been frequently observed in breast cancer [8-19].

Analogous to transcriptomic profiling, DNA methylation profiling is considered to allow the molecular classification of human malignancies and monitoring cancer progression based on a tumor-specific methylation signature [20]. At the same time, it facilitates biomarker discovery for the clinical implementation of this process.

Previous epigenetic analyses have identified aberrant DNA methylation signatures associated with molecular subtypes of breast cancer through hormone-receptor and human epidermal growth factor 2 (HER2) status [21-23]; however, very limited information is available on global methylation changes associated with each molecular subtype, as previous studies focused on individual candidate tumor-suppressor genes by using locus-specific methods. Here we have applied an array-based method [24] for comprehensive DNA methylation profiling to identify differentially methylated genes in breast cancer molecular subtypes. This approach efficiently recognized 15 differentially DNA-methylated loci, which were further validated through pyrosequencing in an independent cohort encompassing 47 basal-like, 44 ERBB2+ overexpressing, 48 luminal A, and 48 luminal B paired breast cancer/adjacent tissues. Our results provide strong evidence for the existence of tumor subtype-specific aberrant methylation profiles, which might be inducers of some transcriptional changes taking place in breast cancer molecular subtypes.

## Materials and methods

### Patients and tumor characteristics

Samples and associated clinicopathologic data were obtained from the Anatomy Pathological Services of the Txagorritxu Hospital, Oncologic Institute of Donostia, and Donostia Hospital (Basque Country). Samples of breast tumor and corresponding adjacent normal-appearing tissue (located at least 2 cm away from the site at which the tumor was sampled) were collected from 215 patients diagnosed with a ductal infiltrative breast carcinoma.

DNA-methylation measurements were performed on DNA isolated from paraffin-embedded primary breast cancer. All breast specimens were reviewed by experienced pathologists. The inclusion criteria were the availability of the paraffin-embedded tissue, tumor size between 1 and 3 cm, histologic grade between 1 and 3, and estrogen receptor (ER)-, progesterone receptor (PR)-, HER2-, CK5/6+, or CK14+, or EFGR+ for the basal-like tumors, ER+, PR±, and HER2+ for the Luminal B, ER-, PR-, and HER2+ for the ERBB2+ tumor group, and ER+, PR+, and HER2- for the Luminal A. Additional data such as Ki-67 status, p53 mutation, and nodal involvement were also registered. Ethical approval for the study was obtained from the corresponding Ethics Committees of the Institutions involved.

### Macrodissection, DNA extraction, and bisulfite modification

To minimize contamination in the methylation analysis, we isolated breast cancer cells and paired normal breast epithelial cells from tissues by manual macrodissection. In brief, 10-μm sections were cut from each archival formalin-fixed paraffin-embedded (FFPE) tissue block. For each pair of tissues, the presence of tumor cells in malignant tissues and the absence of cancer cells in normal tissues were confirmed by histopathologic examination.

A total of 500 μl of buffer TE pH 9 was added to each sample and heated at 100°C for 20 minutes by using a water bath. After heating, a cooling step of 5 minutes was allowed before adding 20 μl of proteinase K. Samples were incubated at 56°C overnight until all the tissue fragments were completely dissolved. Subsequent extraction and purification procedures were performed after the next steps: addition of 500 μl phenol/chloroform/isopropanol alcohol (25:24:1) to the digested tissue, followed by mixing for 10 minutes and centrifugation at 12,000 rpm for 10 minutes. The supernatant fluid was removed to an autoclaved microtube, and one volume of chloroform/isopropanol alcohol (25:1) was added, mixed by vortexing, and centrifuged at 12,000 rpm for 10 minutes. The upper aqueous supernatant was purified by using a DNA purification kit, following the manufacturer's instructions (Qiagen, Valencia, Spain), and the final yield of DNA was dissolved in 50 μl of buffer TE. Sodium bisulfite modification of 1.5 μg DNA was done with the EZ DNA methylation kit (Zymo Research, Orange, CA, USA) by following the manufacturer's protocol.

### Marker discovery study

#### Illumina GoldenGate Methylation Cancer Panel 1

The Illumina GoldenGate Methylation Cancer Panel was used to analyze 550 ng of starting bisulfite-modified genomic DNA. Methylation was represented as a continuous value from 0 (completely unmethylated) to 1 (completely methylated). This value is calculated by subtracting background hybridization levels obtained from negative control probes on the array and calculating the ratio of the fluorescent signal from the methylated allele (M) to the sum of the fluorescent signals from both unmethylated (U) and methylated alleles (|U|+|M|+100).

#### Differential methylation analysis

Microarray data were analyzed to identify the most significant tumor subtype-specific changes relative to the adjacent tissue. Differential methylation was assessed by comparing the mean methylation level (b-value) of samples with the mean b-value of the corresponding adjacent tissue by using BeadStudio (San Diego, CA, USA) and Qlucore Omics Explorer 2.0 (Qlucore AB, Lund, Sweden) software. Selection of the most significantly differentially methylated loci in each tumor subtype was based on (a) Δb value difference of at least 0.20 between the tumors and reference group; (b) an FRD-corrected *P *value cut off of *P *< 0.001, as determined by a two-tailed *t *test [25]; and (c) a *P *value < 0.05 when comparing mean methylation values among the studied tumor subtypes by using analysis of variance (ANOVA) test with Benjamini-Hochberg FDR multiple testing correction. The methylation data have been deposited in NCBI's Gene Expression Omnibus (GEO) [26] and are accessible through GEO Series accession number [GEO:GSE22135].

### Validation of the data by pyrosequencing

#### PCR and pyrosequencing reactions

Selected markers from microarray data analysis were further validated in a larger sample size by bisulfite/pyrosequencing. Additionally, four candidates from the literature were included: *LINE-1*, to measure the global hypomethylation of the tumors [27], and *Let-7a*, *Mir-10a*, and *Mir-93 *microRNAs, because they have been reported to be differently expressed in breast cancer molecular subtypes [28], and their expression might be regulated by methylation in some tumor subtypes, and thus provide new potential subtype-specific biomarkers.

For the methylation analysis, 1.5 μg of genomic DNA was treated with sodium bisulfite, by using the EZ DNA methylation Kit (Zymo Research, Orange, CA, USA) according to the manufacturer's protocol. All primers were designed by using the Assay Design Software (Biotage, Uppsala, Sweden) and synthesized by MWG (Ebersberg, Germany). PCR amplifications were performed by using Qiagen HotStarTaq Master Mix Kit (Qiagen, Valencia, Spain), 7.5 μ*M *biotinylated primer, 15 μ*M *nonbiotinylated primer, and 2 μl of bisulfite-treated DNA (60 ng). PCR primer sequences, PCR conditions, and sequencing primer sequences are given in Table S1 in Additional file 1. The quality and quantity of the PCR product was confirmed by agarose gel (2%) electrophoresis before the cleanup and pyrosequencing analysis. Pyrosequencing was carried out by using the SQA kit (Biotage, Uppsala, Sweden) on a PSQ 96MA Pyrosequencer (Biotage), and the methylation index was calculated by using the Pyro Q-CpG software (Biotage).

#### Validation data analysis

##### Methylation status in tumor versus adjacent tissue

Methylation status was assessed at the studied markers, as previously described by Feng *et al. *[21]. Taking advantage of paired normal/tumor samples, normal tissues' value was considered as the reference. If using the pooled normal samples' mean plus twice the standard deviation as a cut-off point (minimum, 10%), we estimated the probability of the methylation level for a normal-appearing tissue being lower than the cut-off point is <96%. Thus, it is reasonable to assume that samples with a methylation value larger than the cut-off point are likely to be abnormal (or positive). A paired *t *test was used to determine whether a statistically significant change was present in the methylation of the markers examined between the tumors and adjacent tissues. Additionally, to allow the assessment of the observed methylation at multiple promoters as a continuous variable, Z-score analysis was used [29,30]. A Z score for each gene was calculated by using the given formula:

Mean of CpG methylation density of the assessed promoter for each sample - Mean of methylation density for the tumor panel)/SD of methylation density.

A mean Z score was calculated by integrating the promoter-specific Z scores and used as a simple score characterizing mean methylation density. In this analysis, a Z score >0 means methylation greater than the population mean.

##### Comparison between methylation status, breast cancer molecular subtypes, and clinicopathologic characteristic

A one-sample Kolmogorov-Smirnov test was used to evaluate fitness to normal distribution of continuous parameters. Differences in promoter methylation among tumor subtypes were analyzed with ANOVA or Kruskal-Wallis tests as appropriate. The Wilcoxon signed-rank test was used to compare methylation in paired samples. If differences between two independent groups or clinicopathologic characteristics had to be considered, a parametric test (Student *t *test) or nonparametric test (Mann-Whitney *U *test) was used. Comparisons of categoric variables were made by using Fisher's Exact and Pearson's χ^2 ^tests. All reported *P *values are two-tailed and considered statistically significant if *P *< 0.05.

##### Subtype classification

Multivariate logistic regression (MLR) analysis was performed on those biomarkers showing significance in univariate analysis to identify potential biomarker panels capable of discriminating breast cancer subtypes with the best sensitivity and specificity. Models including all possible combinations were constructed and tested by Mallows' Cp selection criterion. The false discovery rate (FDR) of classifying breast cancer subtypes was determined in the best models, and we selected those significant at the FDR <0.2 level.

Supervised hierarchic clustering based on genes selected in the models was performed by using an ANOVA test (Benjamini-Hochberg FDR multiple testing corrected [25]) to confirm results obtained by MLR (Qlucore Omics Explorer 2.0; Qlucore AB, Lund, Sweden). DNA methylation profiles were standardized to have a mean of zero and a standard deviation of 1, and clustering was performed by using the euclidean method and average linkage.

### Ethical considerations

The present study involved analysis of DNA from archival tissue with no subject intervention. No identities were linked to subject records. This study was approved by the Txagorritxu Hospital Review Board under the category of exempt status, and no consent form was required from the participants.

## Results

### Breast cancer subtypes display distinct clinicopathologic characteristics

Patient characteristics are presented in Table 1. Basal-like and ERBB2+ subtypes were more aggressive tumors than luminal A and B ones, as indicated by their histologic and Ki-67 grades (*P *< 0.01), but the basal-like subtype showed less nodal involvement when compared with other subtypes (*P *< 0.01). Additionally, p53 mutation was significantly less frequent in the luminal A subgroup (*P *< 0.05).

**Table 1:** Patient characteristics of both marker discovery and validation studies

		Type			
		
		Basal	ERBB2+	Luminal B	Luminal A
Number of patients		59	49	54	53

Age (years)					
	Mean	56.1	57.1	56.9	62.9
	Median	53	57.5	51	61
	Range (95% CI)	51.5-60.7	52.5-61.6	51.8-61.7	56.9-68.7

Tumor size (cm)					
	Mean	2.4	2.8	3.2	3.1
	Range (95% CI)	2.1-2.7	2.1-3.5	2.5-3.5	2.4-3.7

Histologic grade (%)					
	I	5.0	0.0	0.0	6.0
	II	47.1	24	40.0	55.5
	III	48.9	76	60.0	38.5

Nodal involvement (%)					
	Positive	35.5	63.4	72.2	57.3
	Negative	64.4	36.6	27.8	42.7

Ki-67 overexpression (%)					
	<10%	0.0	8.9	21.9	27.8
	10%-25%	0.0	36.1	31.2	66.7
	>25%	100.0	56.0	46.9	5.6

p53 mutation (%)					
	Positive	47.2	44.1	21.8	14.8
	Negative	52.8	55.9	88.2	85.2

### Array-based DNA methylation-profiling approach identifies some breast cancer subtype-specific methylation changes

**Figure 1:**
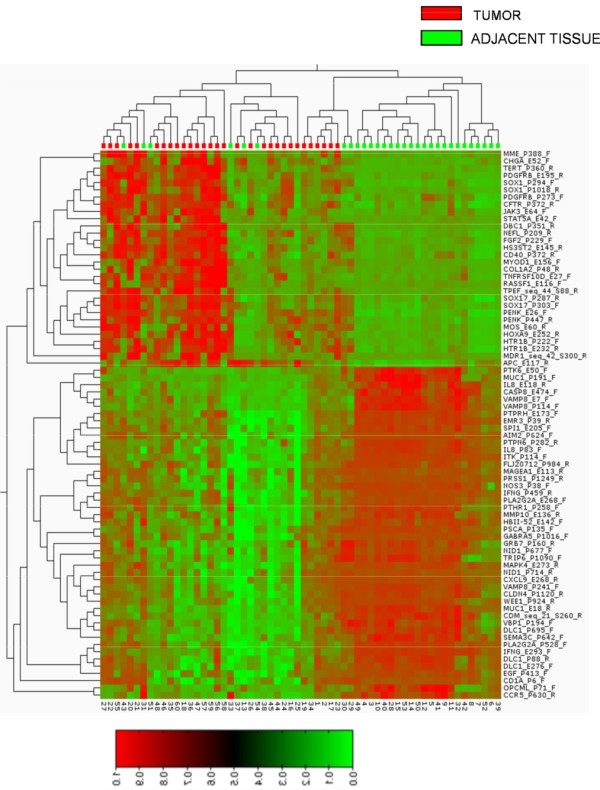
**Supervised hierarchic clustering of 28 paired breast cancer samples by using methylation microarray data from differentially methylated genes**. Heat map and clustering of 76 significant (FRD-corrected, *P *< 0.001) differences between tumors (red) compared with adjacent tissues (green) shows that 30 of the differences (corresponding to 25 genes) are increases of methylation, whereas 47 (corresponding to 37 genes) are decreases of promoter methylation in tumors. Heat-map colors symbolize DNA methylation, as indicated in the color key. The full list of genes is presented in Table S2 in Additional file 2.

DNA methylation-profiling analysis at 1,505 CpG loci was performed in 30 paired breast cancer/adjacent tissues representing the four major breast cancer subtypes. Two of the tumor samples were eliminated from further analysis secondary to their low methylation levels. The final cohort consisted of 60 samples: 28 paired breast cancer/adjacent tissues and four samples from the peritumoral region. Extensive DNA methylation changes between tumor and adjacent tissues were revealed after supervised clustering of the samples (Figure 1; Table S2 in Additional file 2). To identify CpGs with highly significant tumor subtype-specific changes, stringent cut-offs (*P *< 0.001 and Δb >0.20) were set for methylation-level changes relative to controls for each tumor subtype. Distinct CpG loci groups were identified, showing differential methylation profiles in tumor subtypes. We focused in those genes that were significantly differently methylated in the basal-like tumor subtype (*HOXA9, SOX1, VAMP8*, and *TNFRS10D*) and those that were significantly hypermethylated in the luminal B tumors (*NPY*, *RASSF1, HS3ST2, DBC1, FGF2, CD40. SPARC, JAK3, SOX17, PRKCDBP*, and *TAL1*). Additionally, we compared mean methylation values among the studied tumor subtypes by using ANOVA and observed that *NPY, PRKCDBP*, and *RASSF1 *showed lower methylation levels in the basal-like tumor subgroup when compared with other subtypes, and that *DBC1, HS3ST2, FGF2*, and *CD40 *displayed higher hypermethylation levels in the luminal B subtype than in the other subgroups.

The microarray was technically validated by means of pyrosequencing. Comparison of quantitative methylation values at 5 CpG loci by both GoldenGate array and pyrosequencing in 20 samples confirmed the accuracy of the array-based measurements (mean *r*^*2 *^= 0.75; range, 0.56 to 0.91; Table S3 in Additional file 3).

### Validation of differential methylation events in a large panel of breast cancer tissue samples

**Figure 2:**
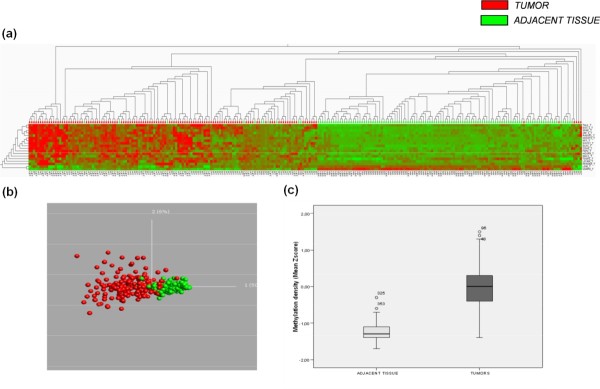
**Supervised hierarchic clustering of 187 paired breast cancer samples by using methylation data from selected genes in the validation study**. **(a)** Heat map and clustering of significant (FRD-corrected, *P *< 0.01) differences between tumors (red) and adjacent tissues (green). Heat-map colors symbolize DNA methylation, as indicated in the color key. **(b) **Principal component analysis (PCA) plots showing separation between tumor and adjacent tissue. **(c) **Box plots showing lower methylation density in adjacent tissue compared with tumors.

For the validation study, pyrosequencing assays for 19 CpG loci were conducted in an independent cohort of 187 normal/breast cancer paired samples encompassing 47 basal-like, 44 ERBB2+, 48 luminal A, and 48 luminal B samples. Differential methylation between tumor and adjacent tissue was confirmed in the validation panel (Figure 2). Among the loci displaying >95% specificity (that is, very low DNA methylation frequency in normal tissue), the frequency of hypermethylation in tumor tissue ranged from 28% (*DBC1*, deleted in bladder cancer 1) to 75% (*JAK3*, Janus kinase 3), (see Table S4 in Additional file 4). Besides, *VAMP8 *(vesicle-associated membrane protein 8) was hypomethylated in 82% of the tumors, and *LINE-1*, the global methylation marker, was highly methylated in all normal tissues (mean = 68% ± 1.9%), whereas a statistically significant decrease was observed in tumors (mean = 63% ± 5.4%; *P *< 0.001).

Further to define those CpG loci differentially methylated in breast cancer subtypes, data were filtered on significance of differences by using the Bonferroni-corrected ANOVA test (*P *< 0.05). We found that 15 of the 19 genes involved displayed different methylation profiles in analyzed breast tumor subtypes (Table 2). *LINE-1, HOXA9, TAL1*, and *SPARC *were excluded, as they showed no significant difference. During the validation study, we confirmed that promoter methylation of *NPY, RASSF1, TNFRS10D, PRKCDBP, Let-7a*, and *VAMP8 *genes was lower in the basal-like subtype when compared with HER2-overexpressing tumors (luminal B and ERBB2+). *SOX1 *and *SOX17 *showed different methylation levels between luminal B and luminal A tumors. We also observed that *HS3ST2, DBC1, FGF2, CD40, JAK3, Mir-93*, and *Mir-10a *markers displayed higher methylation levels in luminal B and ERBB2+ subtypes than in the basal-like and the luminal A tumors. Consequently, the validation study confirmed that these markers were suitable for development of tumor-subtype methylation profiles.

**Table 2:** Methylation levels in breast cancer subtypes

Gene	Methylation level (mean ± SD) (positive rate^a^)				
	
	Basal like	Luminal B	ERBB2+	Luminal A	*P *value^b^
*CD40*	24.3 ± 12.3 (21.7)	30.8 ± 11.9 (36.2)	30.8 ± 11.9 (33.4)	27.4 ± 7.9 (23.9)	<0.001
*DBC1*	12.8 ± 9.2 (15.9)	19.9 ± 10.3 (34.1)	17.8 ± 8.2 (42.7)	14.0 ± 7.3 (19.5)	<0.001
*FGF2*	7.4 ± 7.2 (11.1)	23.1 ± 11.2 (63.8)	32.2 ± 13.9 (67.5)	15.0 ± 13.4 (30.8)	<0.001
*HOXA9*	21.1 ± 12.0 (61.8)	22.5 ± 9.4 (64.7)	28.4 ± 10.3 (83.8)	20.3 ± 8.0 (78.1)	NS
*HS3ST2*	10.3 ± 11.9 (31.0)	23.9 ± 12.9 (75.0)	26.8 ± 12.3 (79.8)	17.5 ± 13.2 (40.4)	<0.001
*JAK 3*	28.0 ± 15.3 (53.5)	36.8 ± 15.2 (87.5)	33.3 ± 13.7 (74.1)	28.8 ± 10.3 (83.0)	<0.001
*Let-7a*	33.9 ± 11.3 (19.1)	55.1 ± 10.9 (72.9)	48.8 ± 15.7 (65.7)	53.5 ± 8.4 (80.0)	<0.001
*LINE-1*	63.8 ± 3.2 (30.2)	63.1 ± 6.2 (40.4)	63.8 ± 7.8 (40.2)	62.8 ± 4.7 (30.4)	NS
*Mir-10a*	15.5 ± 4.2 (36.4)	25.2 ± 5.8 (70.8)	23.1 ± 10.1 (77.4)	20.8 ± 11.3 (63.2)	<0.001
*Mir-93 a*	55.6 ± 7.3 (23.9)	60.6 ± 6.5 (57.8)	61.2 ± 8.9 (62.1)	61.3 ± 6.5 (47.5)	<0.001
*NPY*	14.9 ± 4.9 (35.6)	38.9 ± 14.6 (95.8)	34.5 ± 14.5 (80.9)	27.4 ± 14.8 (78.6)	<0.001
*PRKCDBP*	7.6 ± 6.1 (4.3)	13.2 ± 10.0 (12.5)	15.6 ± 10.1 (22.3)	10.6 ± 8.4 (15.0)	<0.001
*RASSF1*	20.4 ± 11.9 (35.7)	33.9 ± 12.9 (79.2)	35.6 ± 10.8 (74.5)	23.5 ± 5.9 (73.9)	<0.001
*SOX 1*	19.8 ± 10.7 (75.0)	24.5 ± 11.2 (81.3)	21.5 ± 10.1 (78.6)	20.7 ± 10.1 (53.3)	<0.001
*SOX17*	19.1 ± 15.6 (59.1)	22.4 ± 14.2 (70.8)	23.2 ± 14.7 (62.4)	14.1 ± 7.2 (32.4)	<0.001
*SPARC*	35.3 ± 10.7 (48.9)	33.7 ± 10.2 (60.4)	34.5 ± 12.2 (67.3)	31.1 ± 11.0 (68.1)	NS
*TAL1*	38.8 ± 12.5 (59.1)	33.9 ± 11.3 (56.5)	37.7 ± 11.6 (60.4)	27.4 ± 7.9 (52.3)	NS
*TNFRS10D*	12.5 ± 12.6 (27.8)	18.9 ± 8.8 (60.9)	23.5 ± 11.3 (65.7)	19.6 ± 9.2 (60.0)	<0.001
*VAMP8*	8.8 ± 3.8 (93.1)	12.4 ± 4.4 (72.1)	11.4 ± 3.9 (77.2)	11.9 ± 4.1 (79.2)	<0.001

^a^Positive rate using the sample mean plus 2 times the SD of the pooled normal samples (and minimum 10% methylation) as a cut-off point. ^b^*P *value computed by using the ANOVA or Kruskal-Wallis tests, as appropriate.

One of the most interesting aspects of the validation study was to observe that whereas the microarray analysis showed no specific methylation profile for HER2-overexpressing tumors (luminal B and ERBB2+ tumors), the validation study (carried out in a larger sample size) demonstrated that HER2-overexpressing tumors had a common methylation profile.

The analysis of DNA-methylation profiles with regard to the tumors' clinicopathologic features revealed that hypermethylation of *HS3ST2 *was more prevalent in patients with nodal involvement (χ^2 ^= 10.61; *P *< 0.001), whereas *RASSF1 *hypermethylation was associated with the presence of p53 mutations (χ^2 ^= 7.51; *P *< 0.001) and high Ki-67 index (χ^2 ^= 15.63; *P *< 0.001). In addition, promoter hypermethylation of *TAL1 *and *SPARC *genes was more frequent in tumors with high histologic grade (χ^2 ^= 6.41 and χ^2 ^= 6.21, respectively; *P *< 0.05).

**Figure 3:**
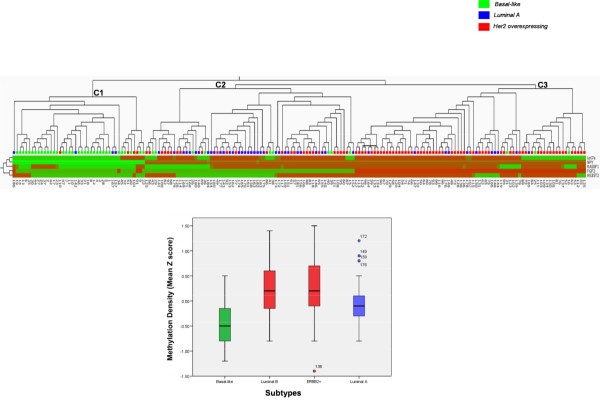
**Supervised hierarchic clustering of the five candidate genes (*Let-7a, NPY, RASFF1, FGF2*, and *HS3ST2*)**. Differential methylation of patterns is shown by red (hypermethylated) versus green (nonmethylated) colors. As noted, three differentiated clusters (C1 to C3) are generated by this statistical analysis. Underneath, box plots showing differential methylation density among tumor subtypes.

We next combined all the methylation data into a single variable, the mean Z score, to allow further analysis. As shown in Figure 2c, mean methylation density in tumors was significantly higher than that in adjacent tissue [-1.26 (CI, -1.26 to -1.17) and -0.01 (CI, -0.81 to 0.72); *P *< 0.001]. Additionally, we found significant differences in the mean Z scores among the different breast cancer subtypes [basal-like, -0.45 (-0.58 to -0.32); luminal B, 0.24 (0.09 to 0.39); ERBB2+, 0.22 (0.05 to 0.41); and luminal A, -0.02 (-0.14 to 0.09); *P *< 0.001] (Figure 3c), and that significantly higher Z-score epigenotypes were related to *LINE-1 *hypomethylated tumors [0.27 (CI, 0.04 to 0.28) and -0.06 (CI, -0.13 to 0.02; *P *< 0.05], HER2-overexpressing breast cancers [0.25 (CI, 0.15 to 0.37) and -0.22 (CI, -0.33 to -0.22); *P *< 0.001], and node-positive tumors [0.11 (CI, -0.01 to 0.19) and -0.10 (CI, -0.25 to 0.4); *P *< 0.05], which are features associated with clinically more-aggressive tumors. Paradoxically, highly proliferative and p53-mutated tumors, both associated with the basal-like phenotype, showed significantly lower Z scores of methylation [-0.17 (CI, -0.35 to 0.01) and 0.11 (CI, -0.03 to 0.26); *P *< 0.001] and [-0.23 (CI, -0.44 to -0.11) and 0.12 (CI, -0.02 to 0.26); *P *< 0.001] respectively.

### Novel DNA methylation profiles with subtype-predictive value

Selected loci were further processed to identify the best biomarker panel allowing classification in tumor subtypes. Multivariate logistic regression (MLR) analysis was performed to identify potential biomarker panels with the best sensitivity, specificity, and false discovery rate (FDR). We first pruned all the variables showing significance in univariate analysis (Table 3) by using all-subset selection with Mallows' Cp optimization criteria, and recognized biomarker panels capable of discriminating basal-like and HER2-overexpressing subtypes at FDR 0.2 level. Specifically, basal-like tumors were inversely associated with hypermethylation of *NPY, FGF2, HS3ST2, RASSF1*, and *Let-7a *markers (sensitivity, 75%; specificity, 95%; and FDR, 0.17), whereas HER2-overexpressing subtypes were strongly correlated to hypermethylation of *NPY, FGF2, HS3ST2, RASSF1*, and *Let-7a *(sensitivity, 74%; specificity, 80%, and FDR, 0.18).

**Table 3:** Subtype-specific methylation profiles in univariate analysis

Tumor subtype	Related genes^a^
Basal-like	* JAK3(-), NPY(-), RASSF1(-), SPARC(-), DBC1(-), FGF2(-), HS3ST2(-), PRKCDBP(-), TNFRS10D(-)*, and *VAMP8(+)*
HER2 overexpressing	* NPY(+), DBC1(+), FGF2(+), RASSF1(+), HS3ST2(+), SOX17(+)*, and *TNFRS10D(+)*
Luminal A	* SOX1(-), SOX17(-)*, and *VAMP8(+)*

^a^Related genes in univariate logistic regression analysis at 0.05 significance level. (+), positively, and (-) negatively related.

The supervised hierarchic-clustering analysis based on these five genes resulted in three different clusters of tumor groups (C1 to C3) (Figure 3). Samples fitting in cluster C1 (characterized by lack of methylation) were mostly basal-like tumors; cluster C2 was the most heterogeneous one with regard to both methylation status of the genes and samples fitting into it. This cluster contains the majority of the luminal A samples, but also includes some luminal B and ERBB2+ tumors. Conversely, cluster C3 (hypermethylated in almost all the analyzed markers) was basically composed of luminal B and ERBB2+ samples (HER2-overexpressing subtypes).

## Discussion

Identification of gene expression-based breast cancer subtypes is considered a critical means of prognostication, and furthermore, an important predictive marker for the response to treatment with endocrine therapy. However, analytic tests relying on RNA measures are difficult to standardize and implement because of the instability of the mRNA transcripts. DNA-methylation profiles reflect phenotypically important differences in gene transcription and, in contrast to most mRNAs, a very stable structure, making DNA-methylation profile-based diagnostic tests highly accurate and reproducible [31]. By use of two independent cohorts of invasive breast carcinomas, our study is, to our knowledge, one of the first to deliver specific methylation profiles associated with basal-like, ERBB2+, luminal A, and luminal B molecular subtypes of breast cancer.

Statistical analysis of the methylation microarray data revealed extensive DNA-methylation changes between tumor and adjacent tissue (Figure 1, Table S2 in Additional file 2) and concurrence of some of these DNA-methylation changes with particular breast cancer molecular subtypes and tumor morphologies. Applying stringent criteria, we identified significant differential methylation among the tumor subtypes in 15 of the 1,505 screened CpG islands. Subsequent validation of the selected genes plus four more from the literature by pyrosequencing in an independent cohort of 187 breast tumor/normal pairs, confirmed that genes tested have significantly altered methylation profiles in comparison to normal-appearing adjacent tissue, and that most of the candidate genes (15 of 19) displayed significantly different methylation profiles between different tumor subtypes. These results corroborate the potential usefulness of the studied markers for the development of a methylation marker panel defining tumor subtypes. Moreover, the current study represents the first evidence for DNA hypermethylation of *NPY, FGF2, CD40, TAL1, JAK3, SPARC, PRKCDBP, DBC1, SOX1, TNFRS10D, Let-7a, Mir-10a*, and *Mir-93 *and hypomethylation of *VAMP8 *in breast cancer.

Several genes have been previously reported to be aberrantly methylated in breast cancer (reviewed in [32-34]). Furthermore, methylation in breast cancer has already been connected to breast cancer molecular subtypes, but these observations require further confirmation. As previously suggested, we found that basal-like, ERBB2+, luminal A, and luminal B molecular subtypes displayed specific methylation profiles. Specifically, HER2-enriched breast tumors (ERBB2+ and luminal B) were associated with the hypermethylation of several genes related to cancer development (Table 3). These results are in accordance with previous studies stating that HER2/neu breast cancers are associated with preferential hypermethylation of several genes [21-23]. In addition, Terada *et al. *[35] recently found that frequent CpG islands methylation is highly associated with HER2 amplification. Conversely, we observed that basal-like tumors were inversely related to promoter methylation of many of the studied genes (Table 3) and showed the lowest methylation levels among the studied subtypes, as indicated by the mean Z-score values (Figure 3c). These findings are consistent with the recent observations made by Holm *et al. *[36], who reported that basal-like tumors have low methylation levels of several CpG sites, whereas luminal B tumors display high methylation levels.

Additionally, our efforts to identify and validate breast cancer subtype-specific epigenotypes resulted in a significant model based on five biomarkers, which is capable of discriminating basal-like and HER2-overexpressing subtypes. Basal-like tumors showed lack of methylation at *NPY, FGF2, HS3ST2, RASSF1*, and *Let-7a *markers, whereas HER2-overexpressing tumors (luminal B and ERBB2+ subtypes) were related to hypermethylation of these markers.

Several authors have speculated that these genomically defined tumor subtypes may represent transformation of stem cells. Some of these hypotheses suggest that mammary stem cells progress to a luminal progenitor state (with an expression pattern similar to that identified in basal breast cancer), which then progresses to differentiated cells with more luminal characteristics [37]. As can be deduced, this hypothesis locates basal-like tumors in an intermediate differentiation step between the mammary stem cells and the more-differentiated luminal subtypes, which would explain their poor prognosis despite response to chemotherapy [38]. Many studies have been conducted to define methylation profiles in human stem cells [39-42]. Likewise, Calvanesse *et al. *[43] compared the methylation status in human embryonic stem cells (hESCs), cancer cell lines, and normal human primary tissues by using the same platform as that in our study. Their finding was consistent with the view that genes aberrantly hypermethylated in cancer (that is, not hypermethylated in normal tissues) were not hypermethylated in hESCs, termed by these authors the classic class A cancer hypermethylated genes. Interestingly, all the CpG sites related to basal-like tumors in univariate regression analysis, with the exception of RASSF1 (Table 3), belonged to this committed class A category, and in a manner analogous to hESCs; they were not hypermethylated in basal-like tumors, nor in normal breast, but they were hypermethylated in the rest of breast cancer subtypes, suggesting that basal-like tumors may share some similarities in their methylation patterns with those of the hESC cell lines. These shared methylation signatures may reinforce the hypothesis of basal-like tumors arising from a mammary stem cell and progressing to differentiated cells with more luminal characteristics (HER2 enriched, luminal A and B). A recent study by Holm *et al. *[36] also supported this hypothesis; they observed that basal-like tumors seem to arise from luminal progenitors in which genes initiating a differentiated luminal cell fate are repressed by other mechanisms than promoter methylation, such as the Polycomb repressive complex 2 (PRC2).

Finally, epigenetic therapy, including the use of demethylating agents and histone deacetylase inhibitors, is now in clinical trials for myelodysplastic syndrome, leukemia, and ovarian and lung cancers [21]. Recent studies have suggested that co-treatment of DNA-methylation inhibitors and histone deceatylases might be an effective form of epigenetic therapy for breast cancer, as the interplay observed between DNA methylation and histone modifications can result in synergistic induction of tumor-suppressor genes [21,44]. Thus, a possibility exists that epigenetic therapy could play an important role in the immediate future of breast cancer treatment. The information on subtype-specific methylation profiles, described in the present study, might promote a better understanding of the epigenetic regulation mechanisms in breast cancer, thereby contributing to the improvement of epigenetic therapy.

## Conclusions

In this study, we used a microarray approach followed by substantial technical and biologic validation to identify specific epigenotypes for particular subtypes in breast cancer. We clearly demonstrate the differences in the methylation profiles of basal-like and HER2-overexpressing tumors and provide evidence to support the utilization of this biomarker for prognostication and therapeutic stratification of patients with breast cancer.

## Abbreviations

ANOVA: analysis of variance; CpG: cytosine-phosphate-guanine; ER: estrogen receptor; ERBB2+: ER-, PR-, and HER2+ tumor subtype; FFPE: formalin fixed, paraffin embedded; FDR: false discovery rate; GEO: Gene Expression Omnibus (GEO); HER2: human epidermal growth factor 2; hESCs: human embryonic stem cells; MLR: multivariable logistic regression; PR: progesterone receptor.

## Competing interests

The authors declare that they have no competing interests.

## Authors' contributions

NGB, TL, AFF, and AAS carried out the methylation assays and helped prepare the manuscript. IG, AV, RR, and IR provided tissues and clinical data. MCA and MJA carried out HER2, CH5/6, CK14, and EGFR1 assays and provided pathologic supports for tissue microdissection. DM and JD performed the statistical analysis. MMP, TL, MFF, MR, and JKF conceived the study and prepared the manuscript. All authors read and approved the final manuscript.

## Supplementary Material

Additional file 1 **Table S1**. PCR primer and sequencing primer sequences and PCR conditions. Click here for file

Additional file 2 **Table S2**. Genes differently methylated that provided optimal distinction between tumor and normal samples (*P *< 0.001 and Δ b >|0.20|). Click here for file

Additional file 3 **Table S3**. Comparison of quantitative methylation measurements at five CpG loci by both GoldenGate array and pyrosequencing. Click here for file

Additional file 4 **Table S4**. Methylation levels in tumors and adjacent tissues. Click here for file
