# Supplementary material for: DNA methylation epigenotypes in breast cancer molecular subtypes
Source: Breast Cancer Res. 2010 Sep 29;12(5):R77. doi: 10.1186/bcr2721 (PMC3096970; doi:10.1186/bcr2721)
Supplement: Additional file 4 — Table S4. Methylation levels in tumors and adjacent tissues. [file bcr2721-S4.DOCX]

**Table S4. Methylation levels in tumors and adjacent tissues.**

| **Methylation levels of 18 genes from pairs of normal and malignant breast tissues** | | | |
| --- | --- | --- | --- |
| **Gene** | **Methylation level (mean ± SD) (Positive rate*)** | | |
|  | **Cancer** | **Adjacent tissue** | **pValue**** |
|  |  |  |  |
| **CD40** | 28.6 ± 11.2(29) | 15.1 ± 8.1(4) | < 0.001 |
| **DBC1** | 11.2 ± 10.1(28) | 1.2 ± 4.1(3) | < 0.001 |
| **FGF2** | 20.4 ± 15.1(43) | 5.2 ± 7.3(6) | < 0.001 |
| **HOXA9** | 22.3 ± 10.7(72) | 5.5 ± 4.2(4) | < 0.001 |
| **HS3ST2** | 20.3 ± 14.2(55) | 1.1 ± 5.2(4) | < 0.001 |
| **JAK 3** | 32.7 ± 15.3(75) | 8.2 ± 6.2(3) | < 0.001 |
| **Let-7a** | 47.7 ± 14.2(53) | 25.1 ± 10.3(3) | < 0.001 |
| **LINE-1** | 63.2 ± 5.4 (27) | 67.5 ± 1.9(5) | < 0.001 |
| **Mir-10a** | 22.3 ± 14.4(50) | 5.2 ± 5.7(4) | < 0.001 |
| **Mir-93 a** | 59.2 ± 10.2(37) | 45.1 ± 8.1(3) | < 0.001 |
| **NPY** | 30.6 ± 20.1(73) | 1.8 ± 5.6(5) | < 0.001 |
| **PRKCDBP** | 5.5 ± 9.5(13) | 0.1 ± 2.9(1) | < 0.001 |
| **RASSF1** | 28.3 ± 13.1(66) | 10.2 ± 5.5(4) | < 0.001 |
| **SOX 1** | 22.7 ± 11.9(72) | 1.5 ± 3.8(2) | < 0.001 |
| **SOX17** | 20.6 ± 15.2(57) | 0.8 ± 4.9(3) | < 0.001 |
| **SPARC** | 31.7 ± 13.5(61) | 12.6 ± 7.1(4) | < 0.001 |
| **TAL1** | 36.2 ± 12.1(57) | 19.1 ± 6.9(4) | < 0.001 |
| **TNFRS10D** | 18.7 ± 11.0(54) | 2.78 ± 5.9(6) | < 0.001 |
| **VAMP8** | 11.1 ± 4.3(82) | 25.4 ± 4.9(5) | < 0.001 |
|  |  |  |  |

*Positive rate using the sample mean plus two times the SD of the pooled normal samples(and minimum 10% methylation) as a cut-off point.

*****p* value computed using the Wilcoxon signed rank test for paired data. SD. standard deviation.**
